# Association between fatty liver disease and risk of microvascular complications in Type-2 diabetes mellitus: A systematic review and meta-analysis

**DOI:** 10.12669/pjms.41.3.11362

**Published:** 2025-03

**Authors:** Jiawei Shao, Mi Zhou, Xiaoqing Xie, Shaobo Lan

**Affiliations:** 1 Jiawei Shao, Department of Hepatology, Affiliated Hospital of Shaoxing University, Shaoxing, Zhejiang Province 421002, P.R. China; 2 Mi Zhou, Department of Dermatology, Affiliated Hospital of Shaoxing University, Shaoxing, Zhejiang Province 421002, P.R. China; 3 Xiaoqing Xie, Department of Hepatology, Affiliated Hospital of Shaoxing University, Shaoxing, Zhejiang Province 421002, P.R. China; 4 Shaobo Lan, Department of Hepatology, Affiliated Hospital of Shaoxing University, Shaoxing, Zhejiang Province 421002, P.R. China

**Keywords:** Type-2 diabetes mellitus, Non-alcoholic fatty liver disease, NAFLD, Neuropathy, Retinopathy, Nephropathy, Microvascular complications, Meta-analysis

## Abstract

**Objective:**

To summarize the existing evidence on the association between non-alcoholic fatty liver disease (NAFLD) and the probability of microvascular complications in Type-2 diabetes mellitus (T2DM).

**Methods:**

PubMed, EMBASE and Scopus databases search (from inception until October 31, 2023) was done for reports with cross-sectional, cohort or case-control design that included adult participants with T2DM and a documented NAFLD status. The selected studies were required to report on at least one microvascular outcome. Studies reporting adjusted associations were included. Random-effects models were used for all analysis. The pooled effect sizes for the associations were reported as odds ratio (OR) with 95% confidence intervals (CI).

**Results:**

Sixteen studies were analysed. T2DM patients with associated NAFLD had similar risk of neuropathy (OR 1.08, 95% CI: 0.97, 1.21), compared to those without NALFD. NAFLD was associated with slightly lower risk of retinopathy (OR 0.86, 95% CI: 0.75, 0.98; N=10, I^2^=82.6%) an increased incidence of nephropathy (OR 1.21, 95% CI: 1.14, 1.29; N=12, I^2^=82.5%), compared to patients with T2DM but no NAFLD.

**Conclusion:**

Diagnosis of NAFLD in patients with T2DM appears to increase the incidence of nephropathy and decrease the risk of retinopathy. Future studies are needed to confirm these observations.

## INTRODUCTION

Non-alcoholic fatty liver disease (NAFLD) represents a range of the condition distinguished by accumulating hepatic steatosis without a history of an excessive alcohol consumption.^1^ It is considered the most common chronic liver condition globally, reflecting the increasing prevalence of sedentary lifestyles and unhealthy dietary habits.^2^ In its milder forms, NAFLD is often asymptomatic, but in severe cases, it may progress to fibrosis, cirrhosis, and even hepatocellular carcinoma, contributing significantly to the burden of liver-related morbidity and mortality.^2^,^3^ Numerous studies show a bidirectional relationship between NAFLD and various metabolic disorders that are on the rise and are currently affecting approximately 25% of the global population.^4^,^5^ Recent research explored possible association of NAFLD with microvascular complications in T2DM with conflicting conclusions.^6^–^8^

While some studies have indicated an elevated risk of microvascular complications, including nephropathy and neuropathy, in patients with T2DM and fatty liver disease, other studies have documented lower risk of diabetic nephropathy and retinopathy.^9^–^12^ This discrepancy in findings may be attributed to variations in study design, patient populations, and the diagnostic criteria used for both NAFLD and microvascular complications. While a narrative review, conducted by Mantovani et al., reported an independent association of NAFLD with chronic kidney disease and distal or autonomic neuropathy,^13^ the data on the possible association between NAFLD and retinopathy are still limited and unclear. A meta-analysis by Song et al that included nine studies did not find significant correlation between NAFLD and the risk of diabetic retinopathy T2DM patients.^14^

Given these gaps in the current understanding, our systematic review aims to provide a comprehensive assessment of the association between NAFLD and the risk of microvascular complications in patients with T2DM. By synthesizing existing evidence, we seek to clarify these relationships and contribute to the growing body of knowledge that may guide future clinical practice and research priorities.

## METHODS

A comprehensive search in PubMed, EMBASE, and Scopus databases was done for reports published up to October 31, 2023. Our search incorporated the following terms: (NAFLD OR fatty liver OR Steatohepatitis OR liver Steatosis OR Visceral Steatosis) AND (diabetes OR diabetes mellitus OR hyperglycaemia OR insulin resistance OR Type-2 diabetes OR prediabetes) AND (nephropathy OR neuropathy OR retinopathy OR microangiopathy OR microvascular complications). The protocol for this review was registered with PROSPERO (CRD42023483580), and adhered to the PRISMA guidelines for the systematic review.^15^

### Inclusion criteria:

Cross-sectional, prospective and retrospective cohort studies, as well as case-control studies; Studies involving adult participants aged 18 or older diagnosed with T2DM and a documented NAFLD status; Studies reporting on at least one microvascular outcome, including neuropathy, retinopathy, or nephropathy; Assessment of NAFLD presence or absence done by acceptable methods; Studies with the comparative group of patients with diabetes mellitus but without NAFLD; Studies, providing adjusted effect sizes, and considering relevant covariates.

### Exclusion criteria:

Case reports, reviews, conference abstracts, and editorials; Studies involving patients under 18 years of age and those focussing on Type-1 diabetes mellitus; Studies without a comparison group or reporting solely on macrovascular outcomes like coronary artery disease or stroke.

### Study selection:

Following the execution of search strategy across three databases and compilation of the initial pool of studies, duplicates were removed. Subsequently, two researchers independently assessed titles and abstracts, and full texts of relevant studies were screened for eligibility. All differences were resolved by discussion.

### Quality assessment, data extraction and statistical analysis:

The Newcastle-Ottawa Scale (NOS) was used to evaluate quality of included papers.^16^ This method assesses studies based on participant selection (e.g., representativeness of cohorts), comparability (e.g., adjustment for confounders), and outcome assessment (e.g., objective or valid measurements, adequacy of follow-up). Each study’s total NOS score could range from 0 to 9, reflecting its overall quality, with higher scores indicating lower risk of bias and greater methodological rigor. Two independent reviewers systematically extracted key details using a standardized form, including study identifier, subject characteristics, sample size, NAFLD assessment method, microvascular complication assessment, and outcomes. Discrepancies were resolved by discussion, or by consulting with a senior reviewer.

### Statistical analyses:

It was done using STATA version 15.0. The pooled effect sizes for the association were reported as odds ratios (OR) with 95% confidence intervals (CI). Heterogeneity across studies was quantified with the I² statistic, where I² values of 25%, 50%, and 75% were interpreted as low, moderate, and high heterogeneity, respectively. A random-effects model was used to consider the variances in baseline characteristics among included studies. Egger’s test and funnel plot inspection assessed publication bias.^17^ P < 0.05 indicated statistical significance.

## RESULTS

**Fig.1:**
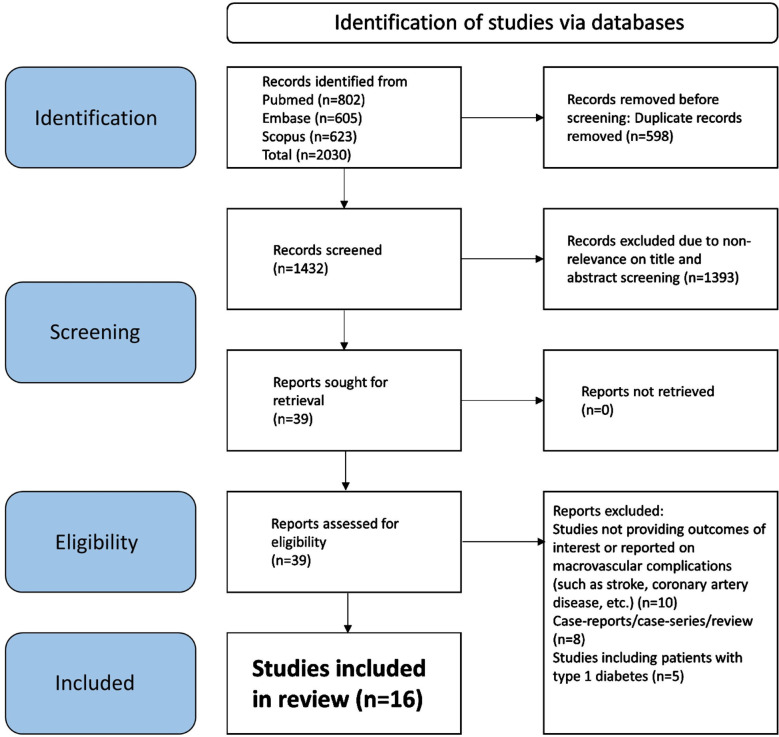
Selection process of studies included in the review.

Our search identified 2030 studies. As shown in Fig.1, after eliminating 598 duplicate papers, 1432 unique studies underwent title and abstract screening. A total of 1393 studies did not meet the predetermined criteria. Subsequently, a detailed assessment of the full texts of 39 studies was done, and 23 more studies were eliminated. Finally, 16 studies were selected for our meta-analysis.^9^–^12^,^18^–^29^ The specific details of the included studies are showed in Supplementary Table-I. Six studies had a prospective cohort design, and another six studies had a retrospective cohort design. Remaining three studies had a cross-sectional design and one was a matched case-control study.

**Supplementary Table-I:** Summary of details of the included studies

Author (year)	Study design; Country	Diagnosis of NAFLD and complications	Sample size	Newcastle Ottawa quality score (NOS)*
Deravi (2023)	Prospective cohort Iran	*NAFLD*: Ultrasound based- in the absence of a secondary cause for steatosis. *Microvascular complications*: Based on International Classification of Diseases, Tenth Revision (ICD-10)	3123 (1215 with NAFLD; 1908 without NAFLD)	8
Rivera-Esteban (2023)	Prospective cohort Spain	*NAFLD:* defined as the presence of steatosis assessed by vibration-controlled transient elastography (VCTE) controlled attenuation parameter (CAP) *Nephropathy:* defined as the presence of microalbuminuria > 30 mg/dL. *Retinopathy:* based on ocular examination	186 (124 with NAFLD; 62 without NAFLD)	7
Wei (2023)	Retrospective cohort China	*NAFLD:* Ultrasound based and in the absence of a secondary cause for steatosis. *Nephropathy:* defined as an eGFR of <60 mL/min/1.73 m^2^ or overt proteinuria.	3627 (2234 with NAFLD; 1393 without NAFLD)	8
Labenz (2022)	Retrospective cohort Germany	*NAFLD and microvascular complications*: Based on International Classification of Diseases, Tenth Revision (ICD-10)	5266 (2633 with NAFLD; 2633 without NAFLD)	8
Seo (2022)	Prospective cohort South Korea	*NAFLD:* Ultrasound based and in the absence of a secondary cause for steatosis. *Nephropathy:* defined as an eGFR of <60 mL/min/1.73 m^2^ for two consecutive times during follow-up visits	3188 (1729 with NAFLD; 1459 without NAFLD)	7
Wen (2022)	Retrospective cohort China	*NAFLD:* Ultrasound based *Retinopathy:* fundal examination or photography *Nephropathy*: urine Microalbumin creatinine ratios ≥30 mg/g or a decrease in eGFR (eGFR<60 mL/min/1.73 m^2^) *Neuropathy*: presence of distal symmetry multiple dysfunctions in neurotransmission of somatosensory evoked potentials	1928 (1181 with NAFLD; 747 without NAFLD)	8
Li (2021)	Prospective cohort China	*NAFLD:* diagnosed by a validated fatty liver index (FLI ≥60) *Nephropathy*: based on eGFR < 60 mL/min per 1.73 m2 and/or a >50% decrease in eGFR from the baseline value	101296 (20,395 with NAFLD; 80,901 without NAFLD)	9
Zhang (2019)	Prospective cohort China	*NAFLD:* Ultrasound based and in the absence of a secondary cause for steatosis *Retinopathy*: based on assessment of fundus images	411 (250 with NAFLD; 161 without NAFLD)	7
Afarideh (2019)	Matched case-control Iran	*NAFLD:* Ultrasound based and in the absence of a secondary cause for steatosis. *Retinopathy*: based on the Early Treatment Diabetic Retinopathy Study (EDTRS) staging system. *Nephropathy:* presence of microalbuminuria, or eGFR <60 mL/min ×1·1.73 m2 *Neuropathy:* Diabetic neuropathy symptom (DNS) score was used	935 (248 with NAFLD; 687 without NAFLD)	8
Yan (2016)	Retrospective cohort China	*NAFLD:* Ultrasound based *Retinopathy:* diagnosed as the presence of retinal hemorrhage, exudates or macular edema *Neuropathy:* presence of persistent numbness, paresthesia, a decreased sense of vibration or a failure to elicit a knee and/or ankle jerk	212 (143 with NAFLD; 69 without NAFLD)	7
Lin (2016)	Cross-sectional USA	*NAFLD:* Ultrasound based and in the absence of a secondary cause for steatosis *Retinopathy*: based on assessment of fundus images	945 (459 with NAFLD; 486 without NAFLD)	7
Jia (2015)	Retrospective cohort China	*NAFLD:* Ultrasound based and in the absence of a secondary cause for steatosis. *Nephropathy:* based on estimation of glomerular filtration rate and urinary albumin excretion rate (UAER)	338 (169 with NAFLD; 169 without NAFLD)	7
Kim (2014)	Retrospective cohort South Korea	*NAFLD:* Ultrasound based and in the absence of a secondary cause for steatosis. *Retinopathy:* evaluated by experienced ophthalmologists *Nephropathy:* based on microalbuminuria, overt albuminuria and azotemia. *Neuropathy:* based on nerve conduction velocity testing or the current perception threshold test.	929 (588 with NAFLD; 341 without NAFLD)	8
Somalwar (2014)	Cross-sectional India	*NAFLD:* Ultrasound based *Retinopathy:* based on dilated fundus examination by an ophthalmologist. *Nephropathy:* presence of positive proteinuria. *Neuropathy:* presence of persistent numbness, paresthesia, a decreased sense of vibration or a failure to elicit a knee and/or ankle jerk	120 (68 with NAFLD; 52 without NAFLD)	7
Viswanathan (2010)	Cross-sectional India	*NAFLD:* Ultrasound based and in the absence of a secondary cause for steatosis *Retinopathy*: dilated fundus examination by Ophthalmologist *Neuropathy*: based on biothesiometry	298 (156 with NAFLD; 142 without NAFLD)	7
Targher (2008)	Prospective cohort Italy	*NAFLD:* Ultrasound based and in the absence of a secondary cause for steatosis. *Nephropathy:* defined as an eGFR of <60 mL/min/1.73 m^2^ or overt proteinuria.	1760 (1289 with NAFLD; 471 without NAFLD)	7

* Each study was evaluated in terms of participant selection (e.g., representativeness of cohorts), comparability (e.g., adjustment for confounders), and outcome assessment (e.g., objective or valid measurements, adequacy of follow-up). Each study’s total NOS score could range from 0 to 9, reflecting its overall quality, with higher scores indicating lower risk of bias and greater methodological rigor.

Majority of the studies were done in China (n=6) followed by Iran (n=2), India (n=2) and South Korea (n=2). One study each was done in the USA, Italy, Spain and Germany. In most studies (n=13), the diagnosis of NAFLD was based on abdominal ultrasound showing presence of hepatic steatosis and supplemented with a history of alcohol use, viral and autoimmune hepatitis or toxicant induced hepatitis (Supplementary Table-I). In the remaining studies, the diagnosis was made either based on International Classification of Diseases, Tenth Revision (ICD-10) codes or fatty liver index (FLI) that considers triglyceride level, body mass index (BMI), waist circumference and gamma- glutamyl transferase level or was defined as evidence of steatosis confirmed by vibration-controlled transient elastography (VCTE) controlled attenuation parameter (CAP) Supplementary Table-I. Methods, used for assessing the outcomes i.e., neuropathy, retinopathy or nephropathy, are summarized in Supplementary Table-I. There was a variability in the assessment methodology and the cut-offs used for defining the outcomes.

This may have implications for the heterogeneity in the pooled effect sizes. Six studies had a NOS score of eight, nine studies had a score of seven and one study had a full score of Nine. The mean score was 7.53, which indicates that the included studies were of acceptable quality. The overall sample size included in our analysis was 1,24,562 patients. Of them, 32,881 were diagnosed with NAFLD and 91,681 comprised the control/comparator (non-NAFLD) group.

### Risk of microvascular complications:

**Fig.2:**
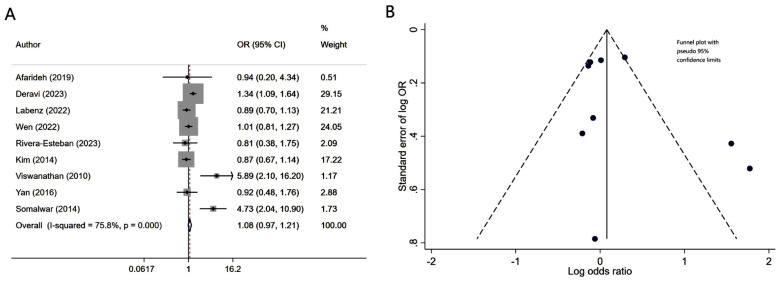
A) Risk of neuropathy in subjects with diabetes mellitus and associated NAFLD, compared to subjects with diabetes mellitus but no NAFLD; B) Funnel plot for bias of neuropathy.

**Fig.3:**
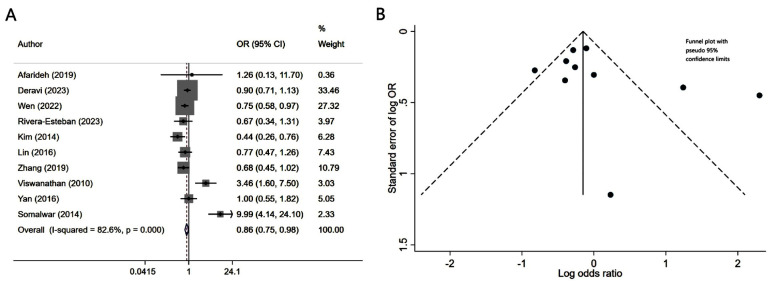
A) Risk of retinopathy in subjects with diabetes mellitus and associated NAFLD, compared to subjects with diabetes mellitus but no NAFLD; B) Funnel plot for bias of retinopathy.

**Fig.4:**
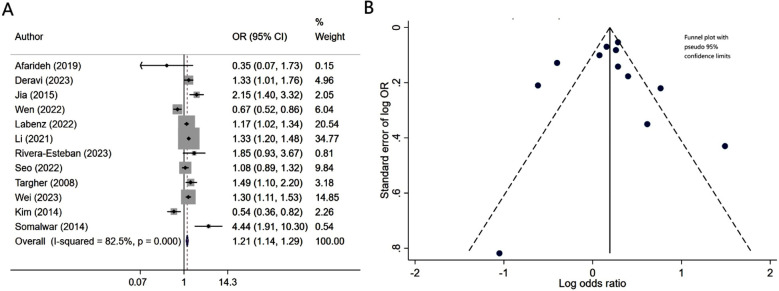
A) Risk of nephropathy in subjects with diabetes mellitus and associated NAFLD, compared to subjects with diabetes mellitus but no NAFLD; B) Funnel plot for bias of nephropathy.

Patients with diabetes mellitus and associated NAFLD had similar risk of neuropathy (OR 1.08, 95% CI: 0.97, 1.21; N=9, I^2^=75.8%), compared to patients without NALFD (Fig.2A). Egger’s test (p-value of 0.37) and visual inspection of funnel plot did not detect publication bias (Fig.2B). However, NAFLD was associated with slightly lower risk of retinopathy (OR 0.86, 95% CI: 0.75, 0.98; N=10, I^2^=82.6%) (Fig.3A). No publication bias was noted for the risk of retinopathy outcome (Egger’s p-value of 0.33; Fig.3B). There was a higher risk of nephropathy in T2DM patients with NAFLD (OR 1.21, 95% CI: 1.14, 1.29; N=12, I^2^=82.5%), compared to T2DM patients with no NAFLD (Fig-4A). Egger’s test (p-value of 0.83) and visual inspection of funnel plot did not detect a publication bias (Fig.4B).

## DISCUSSION

The findings of our meta-analysis indicate that T2DM and concomitant NAFLD correlate with a significantly higher risk of nephropathy, while the risk of neuropathy is comparable to that of patients without NAFLD. At the same time, presence of NAFLD correlated with a slightly lower risk of retinopathy. Our results prompt careful consideration and warrant future exploration to elucidate the underlying mechanisms of these effects.

The observation that patients with T2DM and NAFLD had a slightly lower risk of retinopathy presents an intriguing and somewhat unexpected finding. The association between diabetes mellitus and retinopathy is well-established, and is primarily attributed to prolonged hyperglycaemia, oxidative stress, and inflammation.^30^,^31^ However, the interaction between diabetes mellitus, NAFLD, and retinopathy is a complex interplay that merits careful consideration. It is essential to recognize the limitations of our observation, including potential heterogeneity among the studies, variations in diagnostic criteria for NAFLD and retinopathy, and the observational nature of the data.

Moreover, the relationship between NAFLD and retinopathy may be influenced by confounding factors. The observed lower risk, therefore, could be an artifact of these factors rather than a direct causal relationship. Additionally, a slightly lower risk may not necessarily imply a protective effect of NAFLD against retinopathy. It could be indicative of a more complex interplay of factors that affect the progression of retinopathy in the presence of NAFLD. Further studies, including prospective studies and mechanistic investigations, are needed to confirm the observed association. Numerous studies have demonstrated that T2DM is associated with nephropathy due to prolonged hyperglycaemia, hypertension, and other metabolic factors, associated with T2DM.^32^,^33^ Kidneys are particularly vulnerable to the microvascular complications of T2DM, and diabetic nephropathy is a primary cause of end-stage renal disease.^32^

Our finding of an elevated risk of nephropathy in patients with both T2DM and NAFLD suggests that NAFLD may contribute independently to renal complications beyond the known risk factors associated with T2DM. NAFLD is characterized by hepatic insulin resistance, inflammation, and dyslipidaemia, all of which could potentially impact renal microvasculature and function.^34^,^35^ Several mechanisms may explain the observed association. First, the inflammatory environment associated with NAFLD could contribute to systemic inflammation and endothelial dysfunction, affecting the renal vasculature. Second, insulin resistance, a key attribute of both NAFLD and T2DM, may contribute to impaired renal function. Lastly, the co-occurrence of NAFLD and nephropathy may be related to common genetic and environmental factors.

Our findings highlight the complex interplay between NAFLD and specific microvascular complications in diabetes mellitus. The observed variations in the risk of different complications underscore the need for a tailored and comprehensive understanding of how NAFLD contributes to the diabetic microvascular landscape.

### Limitations:

It include the potential heterogeneity among the included studies, variations in diagnostic criteria for NAFLD and microvascular complications, besides the predominantly observational nature of the studies. Future prospective and clinical studies are warranted to validate our results, and to provide a more robust foundation for clinical implications and potential interventions in patients with diabetes mellitus and NAFLD.

## CONCLUSION

This meta-analysis reveals a complex link between NAFLD and microvascular complications in T2DM patients. While no significant impact of NAFLD on the risk of neuropathy was found, the diagnosis of NAFLD correlated with a slightly lower risk of retinopathy and an increased risk of nephropathy. Our findings highlight the complexity of the interactions between NAFLD and distinct microvascular outcomes. Further research is needed to improve our understanding and refine clinical strategies for this high-risk population.

### Author’s contributions:

**JS:** Concept, study design, literature search, manuscript writing validation and is responsible for the integrity of the study.

**MZ, XX** and **SL:** Data collection, data analysis, interpretation and critical review.

All authors have read and approved the final manuscript.
